# Protective Effect of the Hydrophilic Extract of *Polypodium leucotomos*, Fernblock^®^, against the Synergistic Action of UVA Radiation and Benzo[a]pyrene Pollutant

**DOI:** 10.3390/antiox11112185

**Published:** 2022-11-04

**Authors:** María Gallego-Rentero, Jimena Nicolás-Morala, Miguel Alonso-Juarranz, Elisa Carrasco, Mikel Portillo-Esnaola, Azahara Rodríguez-Luna, Salvador González

**Affiliations:** 1 Department of Biology, Faculty of Sciences, Autónoma University of Madrid (UAM), 28049 Madrid, Spain; 2 Instituto Ramón y Cajal de Investigación Sanitaria (IRYCIS), 28049 Madrid, Spain; 3 Oral and Maxillofacial Surgery Service, Hospital Clínico San Carlos, 28040 Madrid, Spain; 4 Department of Basic Health Sciences, Faculty of Health Sciences, Universidad Rey Juan Carlos (URJC), 28933 Alcorcón, Spain; 5 Department of Pharmacology, Faculty of Pharmacy, University of Seville, 41012 Seville, Spain; 6 Department of Medicine and Medical Specialties, Alcalá de Henares University, 28805 Madrid, Spain

**Keywords:** photopollution, benzo[a]pyrene, ultraviolet A radiation, oxidative stress, *Polypodium leucotomos*, photoprotection, keratinocytes, melanocytes

## Abstract

Oxidative stress is a harmful effect induced on the skin by polycyclic aromatic hydrocarbons (PAH), including benzo[a]pyrene (BaP) air pollutants. This effect is amplified by the additive damaging effect of the sun, especially through the UVA light component. Besides being one of the main compounds that make up air pollution, BaP can also be found in tar, tobacco smoke, and various foods. In addition to its direct carcinogenic potential, BaP can act as a photosensitizer absorbing sunlight in the UVA range and thus generating ROS and 8-hydroxy-2′-deoxyguanosine (8-OHdG). Fernblock^®^ (FB) is an aqueous extract from the leaves of *Polypodium leucotomos* that has been proven to exert photoprotective and antioxidant effects on skin cells. In this study, we evaluate the potential of FB to prevent the damage induced by a combination of BaP and UVA light on human keratinocyte and mouse melanocyte cell lines (HaCaT and B16-F10, respectively). In particular, we have analyzed the capacity of FB to counteract the alterations caused on cellular morphology, viability, oxidative stress and melanogenic signaling pathway activation. Our data indicate that FB prevented cell damage and reduced oxidative stress and melanogenic signaling pathway activation caused by a combination of BaP and UVA light irradiation. Altogether, our findings support the fact that FB is able to prevent skin damage caused by the exposure to a combination of UVA and the air pollutant BaP.

## 1. Introduction

Environmental factors such as sun irradiation (ultraviolet, visible and infra-red components) and air pollutants, influence skin aging and skin diseases, including carcinogenesis. Ultraviolet light (UV) is a recognized carcinogen linked to the induction of both melanoma and non-melanoma skin cancers as well as photoaging [1,2,3,4]. UVC (100–290 nm) and a fraction of UVB (290–320 nm) wavelengths are absorbed by the Earth’s ozone layer, whereas the rest of UVB and UVA (320–420 nm) wavelengths are transmitted through the atmosphere. Both UVB and UVA are responsible for inducing dimerization of pyrimidine bases [5,6,7], thus leading to DNA damage, either directly or indirectly by promoting the production of reactive oxygen species (ROS). The most frequent DNA alterations associated with UV radiation are the formation of cyclobutane pyrimidine dimers (CPDs) [5,6,8]. The dimerization of pyrimidine bases can occur during the irradiation process (light-CPDs) or even several hours after irradiation (dark-CPDs) [1,8].

On the other hand, the World Health Organization (WHO) indicated in 2018 that nine out of 10 people breathe in high levels of air pollutants, with the deaths per year due to air pollution going up to 4.2 million in 2016 [9,10]. Among the air pollutants considered by WHO, polycyclic aromatic hydrocarbons (PAH), including benzo[a]pyrene (BaP), are of particular interest, with a specific group (Working group on PAH) dedicated to evaluate their impact on human health [11]. Particulate matter, nitrogen dioxide, sulfur dioxide, black carbon, carbon monoxide, and ground-level ozone, among others, are also included as air pollutants [10,12]. In addition, the impact of air pollutants in the skin is also under extensive investigation [13,14].

BaP is formed by incomplete combustion of organic matter at high temperatures and is one of the main compounds that make up air pollution. It can also be found in tar, tobacco smoke, and various foods and can exert direct carcinogenic effects by damaging the DNA. In addition, BaP and its intermediates can act as photosensitizers when absorbing sunlight in the UVA range. The transition towards a higher energy excited state leads to the generation of ROS and 8-hydroxy-2′-deoxyguanosine (8-OHdG). 8-OHdG is an oxidative derivative of guanosine formed as a consequence of UV radiation and considered a biomarker of nucleic acid oxidation [15,16].

In fact, in combination with UVA radiation, non-toxic concentrations of PAH can induce increased cell damage in vitro and tumorigenicity in mice [17,18]. Studies performed in normal human epidermal keratinocytes have confirmed that photo-pollution is more dangerous than pollution alone [19]. When administered following the exposure to UVA, PAH decreases cell viability and the levels of phosphorylated histone H2AX (γH2AX), a marker of DNA double-strand breaks [17,20]. UVA accelerates the cellular metabolism of BaP, leading to increased generation of BaP derivatives and consequently to DNA oxidative damage [16]. This induces alterations in pathways such as JNK signaling as well as in the expression of genes involved in cell survival, extrinsic aging, and photocarcinogenesis [21,22]. In addition, the normal epidermal cell cycle can also be altered in skin chronically exposed to photo-pollution. Also, the combination of PAH and UVA can induce mitochondrial damage by triggering the generation of superoxide anion and membrane depolarization [19]. Moreover, the release of cytochrome C from the mitochondria to the cell cytoplasm has also been related to this combination, constituting an important marker of the cytotoxicity derived from the deleterious synergistic action of BaP and UVA light. Indeed, liberation of cytochrome C is a key process for the induction of apoptosis [23,24].

UVA light also has a role in the expression of the photoreceptor opsin-3, related to photoaging in dermal fibroblasts and melanogenesis. Opsin-3 is expressed not only in the eyes but also in the skin. This receptor mediates responses triggered by both visible and UV light. Importantly, this photoreceptor is involved in the regulation of melanogenesis in skin melanocytes [25,26,27]. According to this, previous studies have reported an induction of melanogenesis when opsin-3 is activated by blue light, triggering the activation of the enzyme tyrosinase. However, no studies have addressed the effect of the combination of UVA and PAH on the expression of opsin 3 [28,29].

Previous studies using mice have linked the sequential treatment with BaP and UVA light with the induction of DNA damage, ultimately causing carcinogenesis. Although the independent effect of each agent separately did not increase the number of tumors formed, the sequential treatment with BaP and UVA light generated intermediate metabolites such as BaP t-4,5-diol or BaP t-9,10-diol, leading to DNA damage and mutagenic activity by enhancing the JAK2/STAT3 signaling pathway [30]. In addition, increased rates of non-melanoma skin cancer are found in smokers and the combination of sunlight and tobacco is a risk factor for dysplastic and malignant lip lesions [19,20,31].

Therefore, the hazardous effects of air pollutants on the skin are amplified by the action of the sun, especially UVA radiation. Thus, cutaneous protection strategies that include the use of sunscreen with high capability to absorb UVA light and containing antioxidants can provide greater resistance to oxidative stress in the skin [21,31].

Various botanicals endowed with antioxidant activity are emerging as new natural photoprotective compounds, including *Curcuma longa* extract and trans-resveratrol, the latter being a highly used antioxidant in sunscreens [31,32,33]. In this context, the natural extract from *Polypodium leucotomos* (Fernblock^®^, FB) has been shown to exert antioxidant and photoprotective effects against UVA light in keratinocytes and fibroblasts, the two main cellular components of the skin [33,34,35,36,37]. Additionally, it leads to decreased oxidative damage by enhancing antioxidant cellular mechanisms in skin cells upon exposure to fine pollutant particles (PM_2.5_) [38]. Moreover, it can reduce UVB-induced light and dark-CPDs [39].

In the present work, we investigate the potential effect of FB to prevent cellular damage induced by the sequential treatment with BaP and UVA light in keratinocyte and melanocyte cell lines. In particular, we have analyzed the protective effects of FB on cell morphology, viability, oxidative stress and melanogenic signaling pathway activation. Our results indicate that FB can prevent cellular damage as well as reduce oxidative stress and melanogenic signaling activation caused by sequential exposure to BaP and UVA light. Overall, our findings support the fact that FB can counteract the deleterious synergistic effect of these two environmental agents in the skin.

## 2. Materials and Methods

### 2.1. Cell Culture

The established non-tumorigenic human keratinocyte cell line HaCaT (Cell Line Service, Eppelheim, Germany) and the mouse melanocyte cell line, B16-F10 (provided by Dr. Benilde Jiménez Cuenca, Instituto de Investigaciones Biomédicas “Alberto Sols” UAM-CSIC) were used in the study. Both cell lines were cultured in Dulbecco’s Modified Eagle Medium (DMEM) supplemented with 10% (*v*/*v*) fetal bovine serum (FBS), 1% (*v*/*v*) penicillin G (100 U/mL), and streptomycin (100 mg/mL), all from Thermo Fisher Scientific Inc. (Rockford, IL, USA). Cells were grown under standard conditions at 37 °C, 5% humidity, and 5% CO_2_. Passages of both cell lines were carried out using 1 mM EDTA/0.25% Trypsin (*w*/*v*) (Thermo Fisher Scientific Inc. (Rockford, IL, USA).

### 2.2. UVA Light Irradiation

Keratinocytes and melanocytes were irradiated using a CAMAG UV lamp (CAMAG, cat. no. 022.9115, El Prat de Llobregat, Spain). Cells were subjected to irradiation with UVA for 1, 3, 5, 7 and 10 min, corresponding to doses of 94, 282, 470, 658 and 940 mJ/cm^2^, respectively. For irradiation, the culture medium was replaced with PBS (Thermo Fisher Scientific Inc., Rockford, IL, USA). Cells were placed at a distance of 11 cm under the UVA light source inside a dark box. Immediately after UVA exposure, PBS was discarded and fresh medium was added.

### 2.3. Combined Treatment with BaP (+/−FB) and UVA Light Exposure

The cellular effects of combining BaP and UVA light were tested in both cell lines. First, cells were incubated with BaP (2 µM for HaCaT cells and 5 µM for B16-F10 cells) during 48 h, either in the simultaneous presence or absence of FB. Immediately after incubation, cells were subjected to irradiation with UVA light. For the latter, the medium containing BaP was discarded replaced with the minimum volume of PBS that covered the monolayer of cells, to minimize light refraction the irradiation step. After irradiation, PBS was replaced with freshly prepared complete medium.

### 2.4. Cell Viability Assessment

Cell viability was evaluated 24 h after treatment by using the MTT assay. For this purpose, a stock solution of 1 mg/mL MTT (3-(4, 5-dimethylthiazol-2-yl)-2, 5-diphenyltetrazolium bromide) (Sigma-Aldrich, St. Louis, MO, USA) in PBS was diluted in complete medium to reach a final concentration of 50 μg/mL. The incubation with MTT solution was performed during 3 h under normal culture conditions (37 °C, 5% CO_2_). Then, MTT solution was removed and DMSO (Panreac, Barcelona, Spain) was added to dissolve the formed formazan crystals. The absorbance was measured at 542 nm by using a SpectraFluor Tecan (Zürich, Switzerland) plate reader. The results are presented as the percentage of cell survival. Data obtained from non-treated (control) cells were used as a reference for normalization.

### 2.5. Reactive Oxygen Species Determination

ROS production was determined by fluorescence microscopy using the 2,7-dichloro-dihydrofluorescein diacetate (DHF-DA) probe (Abcam, Cambridge, UK). Cells were incubated for 48 h with BaP or with BaP + FB. Afterwards, cells were incubated with 7.5 M DHF-DA for 30 min under normal culture conditions and they were subsequently irradiated with UVA light. Untreated cells and cells treated with BaP and BaP + FB in the absence of UVA light were used as controls. To assess the levels of ROS, cells were observed under the fluorescence microscope using blue excitation and the fluorescence intensity was quantified using the ImageJ software (version 1.8.0) (NIH, Bethesda, MD, USA).

### 2.6. Indirect Immunofluorescence

For indirect immunofluorescence (IF), cells were grown on glass coverslips and subjected to the treatments indicated above. Then, cells were fixed in formaldehyde for γH2AX and cytochrome C determination or in a mix of a 1:1 proportion of methanol-acetone (Panreac, Barcelona, Spain) for 8-OHdG determination. Fixation with 3.7% formaldehyde (Sigma-Aldrich, St. Louis, MO, USA) in PBS was carried out at 4 °C for 30 min. Afterwards, cells were washed three times with PBS and permeabilized with 0.5% Triton X-100 in PBS at room temperature. For 8-OHdG, after fixation cells were treated with 0.05 N HCl and sequentially washed with PBS and 35%, 50% and 70% ethanol. DNA denaturation was carried out with 0.15 N NaOH. Cells were again washed with 70% diluted in 4% formaldehyde, 50% and 35% ethanol and PBS.

Coverslips were subsequently incubated with the primary antibodies against γH2AX (Cell Signaling Technology, Inc., Danvers, MA, USA), cytochrome C (Invitrogen, Waltham, MA, USA) or 8-OHdG (Abcam, Cambridge, UK) diluted in 0.5% bovine serum albumin (BSA, Sigma-Aldrich, St. Louis, MO, USA) in PBS for 1 h at 37 °C. After washing with PBS, cells were incubated with the secondary antibodies AF546 goat anti-rabbit IgG or AF488 goat anti-mouse IgG (Thermo Fisher Scientific Inc. (Rockford, IL, USA)) for 45 min at 37 °C. Afterwards, cells were washed again, and nuclei were counterstained with Höechst-33258 (Sigma-Aldrich, St. Louis, MO, USA) for 5 min at 37 °C. Finally, the coverslips were washed in PBS and mounted with ProLong (Thermo Fisher Scientific Inc. (Rockford, IL, USA). The slides were observed using an epifluorescence microscope (Olympus BX61) equipped with a HBO 100 W mercury lamp and the filter sets for fluorescence microscopy, blue light irradiation (450–490 nm, BP 490 filter) for cytochrome C and 8-OHdG, ultraviolet irradiation (360–370 nm, UG-1 filter) for nuclei and green light irradiation (570–590 nm, DM 590 filter) for γH2AX. For the quantification of the intensity of 8-oxo-dG signal, the ImageJ software (version 1.8.0) (NIH, Bethesda, MD, USA) was used. 

### 2.7. Mitochondrial Morphology

In order to assess the effects of treatments on mitochondrial morphology, the 3,3′-Dioctadecyl-5,5′-Di(4-Sulfophenyl)Oxacarbocyanine (DIOC) green fluorescent probe (Invitrogen, Thermo Fisher Scientific, Waltham, MA, USA) was used. Cells were seeded on coverslips and treated for 48 h as indicated above, followed by incubation with 5 μM DIOC for 30 s at 37 °C before irradiation with UVA. Untreated cells, as well as those exposed to single treatment with BaP or UVA, were incubated with DIOC and used as controls. After the incubation with DIOC, cells were washed with PBS and observed using phase contrast or fluorescence microscopy under blue light excitation (450–490 nm, BP 490 filter) with an epifluorescence microscope (Olympus BX61) equipped with a HBO 100 W mercury lamp.

### 2.8. Quantification of the Expression of Opsin-3 by Real Time Polymerase Chain Reaction

Expression of opsin-3 mRNA was determined by Real-Time Polymerase Chain Reaction (RT-PCR). To this end, B16-F10 cells were incubated with BaP, FB or the combination of both for 48 h prior to irradiation with UVA. After 24 h since the irradiation with UVA light, cells were washed with PBS, scraped, and centrifuged. Supernatant was discarded and RNA was extracted from the pellet using a mini-RNeasy kit (Qiagen). Concentration and purity of the RNA samples were determined by spectrophotometry (NanoDropND1000, Nanodrop Technologies, Wilmington, DE, USA). RT-PCR was performed with the corresponding Taqman probe (Mm00438648_m1, Thermo Fisher Scientific Inc. (Rockford, IL, USA) and the results were analyzed using the delta-delta-cycle threshold method (ddCt) [40].

### 2.9. Statistical Analyses

All the experiments were repeated at least three times. The GraphPad Prism software (GraphPad Software Inc., San Diego, CA, USA, version 6.05) was used to perform the statistical analysis (one-way ANOVA or *t* test) and data representation. Statistical significance was set at *p* < 0.05.

## 3. Results

### 3.1. Characterization of the Photoprotective Effects of FB on Cell Viability and Morphology

We first evaluated the independent effects of BaP and UVA light on cell lines representative of the main epidermal cellular components, keratinocytes and melanocytes. Cell survival after the treatments was assessed using the MTT assay. At the administered doses, UVA light alone did not induce any toxicity when compared to unexposed (control) cells (Figure S1a). Similar results were observed upon treatment with different concentrations of BaP (Figure S1b).

**Figure 1:**
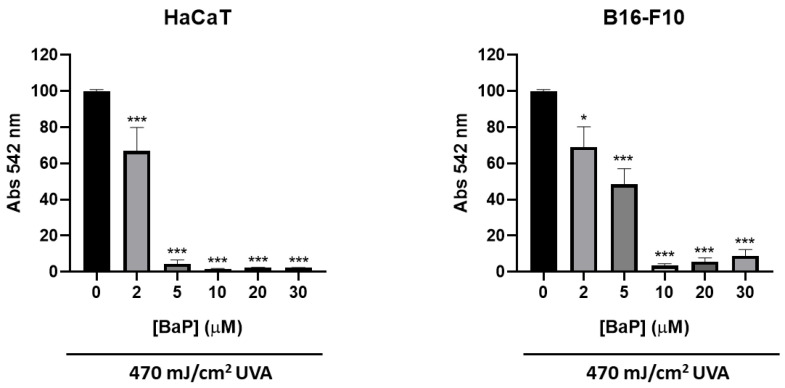
Effect of BaP plus UVA in HaCaT and B16-F10 cell lines. Cells were treated with different concentrations of BaP (2, 5, 10, 20 and 30 µM) for 48 h and immediately exposed to a dose of 470 mJ/cm^2^ of UVA light. The results obtained from MTT assay revealed a decrease in cell survival as BaP concentration increased. Error bars denote ± S.E.M. (*n* = 3, one-way ANOVA *: *p* < 0.05; ***: *p* < 0.001).

In order to study the cytotoxic effect of the sequential combination of BaP and UVA light on skin, we proceeded to expose the cells to the combined treatment with both agents. Interestingly, the treatment with BaP for 48 h (at doses of 2, 5, 10, 20 and 30 µM) followed by immediate exposure to 470 mJ/cm^2^ of UVA light led to a decrease in cell survival in both melanocytes and keratinocytes (Figure 1). Concentrations of 2 µM and 5 µM of BaP were selected for the combination assays in HaCaT and B16-F10 cells, respectively, since those concentrations induced a 50% of cell lethality (LD50) when combined with 470 mJ/cm^2^ of UVA light.

We next evaluated the capacity of FB to prevent the harmful effects of the combination of BaP and UVA. First, we tested the effect on cell survival of different FB concentrations (0.001–0.1 mg/mL) in combination with the selected BaP concentrations but in the absence of UVA light. The results indicated that the combination of BaP and FB induced no cytotoxic effects (Figure S2). Based on these results and on previous studies performed in our laboratory [37,41,42], 0.01 mg/mL was selected as the FB concentration to assess the photoprotective potential of this compound.

**Figure 2:**
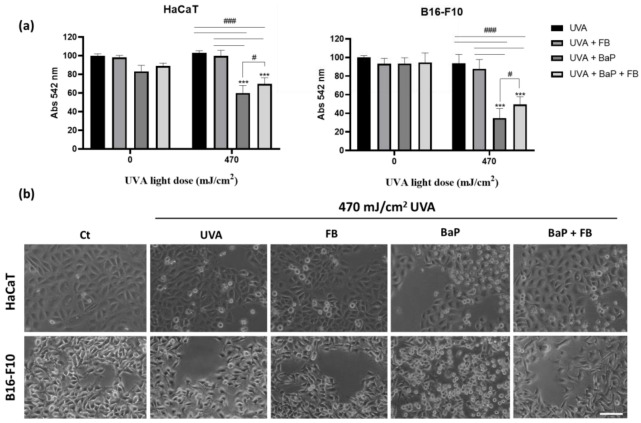
Effects of BaP, FB and UVA light exposure on the viability and morphology of HaCaT and B16-F10 cell lines. (**a**) Cell viability rates of both cell lines treated with BaP (2 µM, HaCaT; 5 µM, B16-F10), FB (0.01 mg/mL) and exposed to 470 mJ/cm^2^ of UVA light. The results of the MTT assay indicated that the treatment with FB exerted a photoprotective effect in both cell lines. (**b**) Phase-contrast microscopy images illustrating the changes in cell morphology after the treatments. When treated with FB, a reduction in cell death could be observed compared to cells treated only with BaP. Error bars denote ± S.E.M. (*n* = 3, one-way ANOVA ***: *p* < 0.001; and *n* = 3, *t*-test #: *p* < 0.05; ###: *p* < 0.001). Scale bar = 50 µm.

Cells were incubated with a mixture of BaP (2 µM for HaCaT and 5 µM for B16-F10 cells) and 0.01 mg/mL FB for 48 h and subsequently irradiated with UVA light. The results revealed no significant photoprotective effects of FB in any of the cell lines upon exposition to BaP followed by irradiation with 282 mJ/cm^2^ (Figure S3). However, FB efficiently exerted photoprotection on cells pre-treated with BaP and subsequently exposed to 470 mJ/cm^2^ of UVA (Figure 2a). In addition, we evaluated the morphological changes (cell retraction and rounding) by phase-contrast microscopy upon the different treatments. The cell cultures incubated with FB or BaP, either alone or in combination, displayed no vast morphological changes, basically retaining the morphology of untreated keratinocytes and melanocytes (Figure 2b). However, substantial changes in cellular morphology were observed in the cells pre-treated with BaP and exposed to UVA irradiation. We observed cell retraction, blebs’ formation as well as cell rounding in both cell types, all related with cell death. The treatment with FB partially prevented the induction of morphological alterations in response to the sequential treatment with BaP and UVA.

### 3.2. Evaluation of Oxidative Stress Levels

**Figure 3:**
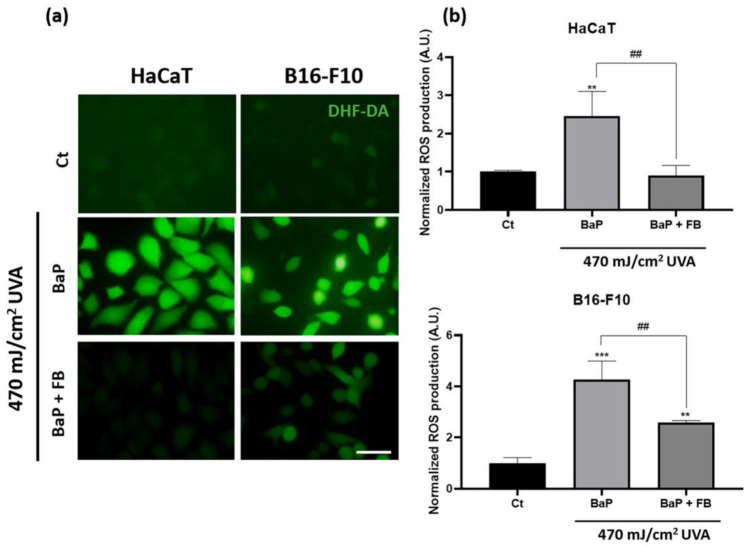
Reactive oxygen species generation in HaCaT and B16-F10 cells with BaP and FB treatment followed by UVA light exposure. (**a**) Cells were incubated with BaP and BaP + FB, followed by DHF-DA incubation. Subsequently, cells were irradiated with 470 mJ/cm^2^ of UVA light and observed under the fluorescence microscope using blue light excitation. Green fluorescence revealed ROS production. Cells treated with BaP and UVA showed the highest fluorescence intensity, while cells treated with both BaP and FB prior to UVA irradiation diminished ROS levels. Scale bar: 40 µm. (**b**) ROS production was quantified using the ImageJ software. Cells treated with BaP and UVA light showed a significant increase in ROS generation when compared to control cells. Oxidative stress in cells treated with BaP and FB decreased significantly when compared to BaP + UVA in both cell lines. Error bars denote ± S.E.M. (*n* = 3, one-way ANOVA **: *p* < 0.01; ***: *p* < 0.001; and *n* = 3, *t*-test ##: *p* < 0.01).

To evaluate ROS production induced by the treatments, after the incubation with BaP or BaP + FB, cells were incubated with DHF-DA before the irradiation with 470 mJ/cm^2^. The results revealed significantly increased ROS levels in both cell lines treated with BaP and exposed to UVA light when compared to non-treated (control) cells. However, FB effectively prevented the increase in ROS production derived from the exposure to BaP and UVA light in both cell lines, hence suggesting that FB could reduce the oxidative stress-induced damage derived from the treatment with the combination of both agents (Figure 3a,b). No changes in ROS production were observed in cells exposed to single treatments (only BaP or only UVA) (Figure S4).

### 3.3. Mitochondrial Morphology and Localization of Cytochrome C

**Figure 4:**
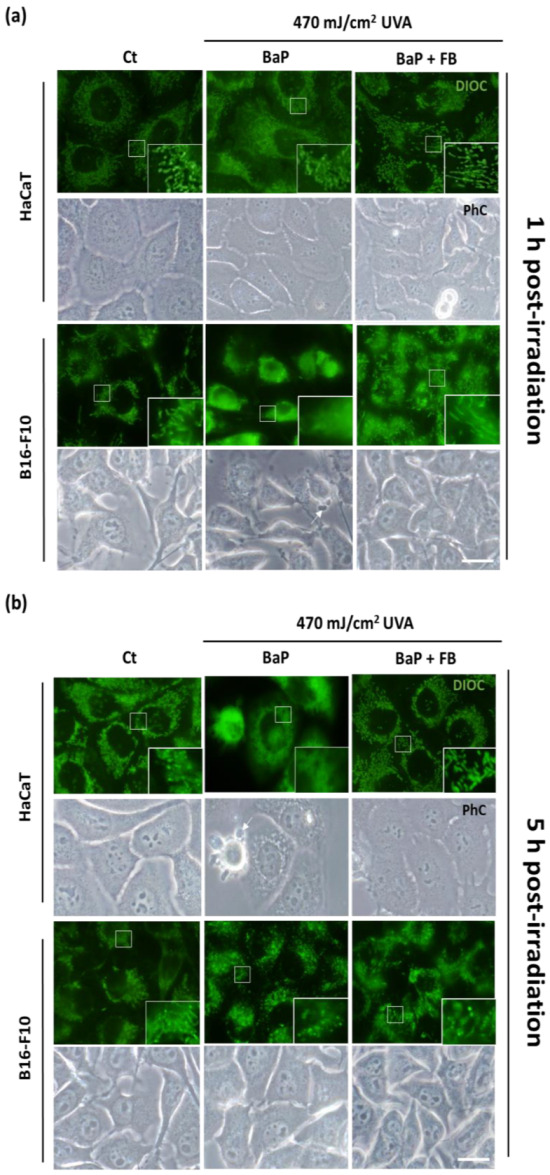
Mitochondrial morphology in HaCaT and B16-F10 cells exposed to BaP, FB and by the combination with UVA light irradiation. Cells were loaded with the DIOC probe BaP and BaP + FB treatments before irradiation. (**a**) Mitochondrial morphology 1 h after irradiation. A spherical appearance showed in cells treated with BaP. Mitochondria of cells treated with BaP and FB maintained the control filamentous morphology. (**b**) Mitochondrial morphology 5 h after irradiation. Mitochondria of cells loaded with BaP appeared with a spherical morphology, while cells treated with BaP and FB retained the normal morphology. Phase contrast images did not show morphological changes in cell shape except for those treated with BaP, appearing some of them with small blebs due to cytotoxic effects of the treatments (arrows). Scale bar: 20 µm.

To assess the effects on mitochondrial morphology, immediately after the treatment with BaP or BaP + FB the cells were loaded with the DIOC fluorescent probe prior to irradiation with UVA light (470 mJ/cm^2^). At different time points (1 h and 5 h) after irradiation with UVA, mitochondrial morphology was evaluated under blue light excitation. Control cells displayed filamentous mitochondrial morphology, especially in the B16-F10 cell line (Figure 4a,b). In response to the treatment with BaP followed by irradiation with UVA light, we observed changes in mitochondrial morphology in both cell lines. Mitochondria became more rounded 1 h after irradiation (Figure 4a) and displayed signs of severe damage 5 h after irradiation, showing a retracted and rounded morphology (Figure 4b). On the contrary, the treatment with FB in combination with BaP followed by UVA irradiation prevented this morphological change in mitochondria, which retained the filamentous shape (Figure 4a,b). These results pointed to a protective effect of FB (Figure 4a,b). Moreover, morphological changes were only observed by phase contrast microscopy in cells treated with BaP and UVA irradiation, displaying blebs (Figure 4a,b). Cells treated with only BaP or only UVA light did not show morphological changes in their mithocondria (Figure S5).

**Figure 5:**
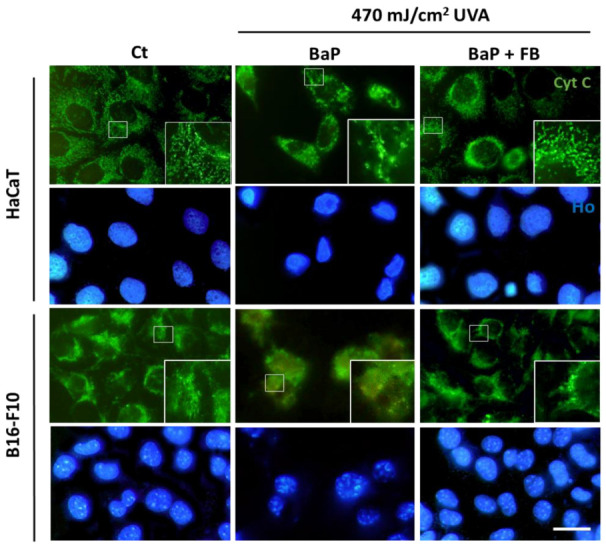
Effect of BaP and FB exposed to UVA light on the dynamics of cytochrome C in HaCaT and B16-F10 cells. Cells were incubated with BaP and BaP + FB and immediately after exposed to 470 mJ/cm^2^ of UVA light. 24 h after irradiation, cytochrome C localization was evaluated. The results showed altered mitochondrial morphology and cytochrome C release in cells treated with a combination of BaP and UVA light. In both cell lines, cells treated with BaP + FB followed by UVA light irradiation showed heterogeneity in cytochrome C localization. Some cells retained a normal mitochondrial morphology, while others released the molecule to the cytoplasm. This suggests a partial protective effect of FB. Scale bar: 20 µm.

Cytochrome C is a protein located in the intermembrane space of the mitochondria. When apoptotic processes are activated, this molecule can be released into the cytoplasm [24]. The localization of cytochrome C in response to the treatments was evaluated by fluorescence microscopy 24 h after UV radiation. Our results indicated that the altered mitochondrial morphology observed in cells treated with BaP and subsequently exposed to UVA light were linked to cytochrome C release into the cytoplasm (Figure 5). In support to the combined role of these two agents, cytochrome C retained its typical localization within the mitochondrial intermembrane space in response to single treatment with either BaP or UVA light (Figure S6). Interestingly, the addition of FB during the incubation with BaP prior UVA light exposure partially restored the localization of cytochrome C, suggesting that FB can act as a protective agent in both cell lines.

### 3.4. Evaluation of DNA Damage

**Figure 6:**
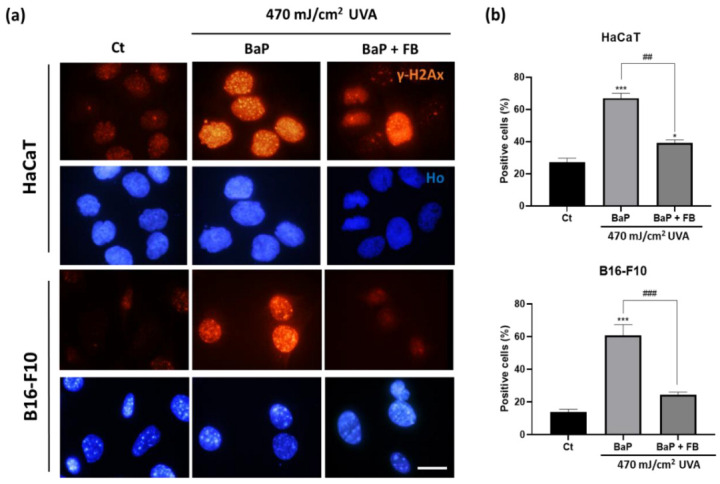
γH2AX localization and positiveness in HaCaT and B16-F10 cells after treatments. Cells incubated with BaP or BaP + FB for 48 h and immediately exposed to 470 mJ/cm^2^ of UVA light. γH2AX expression was evaluated 24 h after light exposure. (**a**) Fluorescence microscopy images of positive and negative γH2AX cells. Cells treated with BaP and then exposed to UVA light displayed increased γH2AX expression levels. Cells treated FB + BaP and subsequently exposed to UVA showed a similar percentage of γH2AX-positive nuclei at in the case of control cells. Scale bar: 20 µm. (**b**) Quantification of positive γH2AX cells in both cell lines. The results showed a significant increase in cells treated with BaP and exposed to UVA light. However, cells treated FB during BaP exposition prior to UVA light showed a significant decrease of positive cells. Error bars denote ± S.E.M. (*n* = 3, one-way ANOVA *: *p* < 0.05; ***: *p* < 0.001; and *n* = 3, *t*-test ##: *p* < 0.01; ###: *p* < 0.001).

γH2Ax is a histone protein from the H2A family that undergoes phosphorylation at the Ser139 residue early upon the appearance of DNA double strand breaks [43]. We evaluated the presence of positive nuclei for the expression of the phosphorylated form of this histone (γH2Ax) by immunofluorescence in response to the different treatments. The percentage of γH2Ax positive cells was significantly increased in HaCaT and B16-F10 cells upon treatment with BaP followed by UVA irradiation, compared to untreated cells. On the contrary, neither BaP alone nor UVA light alone induced significant changes in the percentage of γH2AX positive nuclei in comparison with the control (Figure S7). It should be noted that addition of FB during the incubation with BaP and prior to UVA exposure significantly reduced the percentage of γH2AX positive cells, reaching values similar to those observed in the control condition (Figure 6a,b).

**Figure 7:**
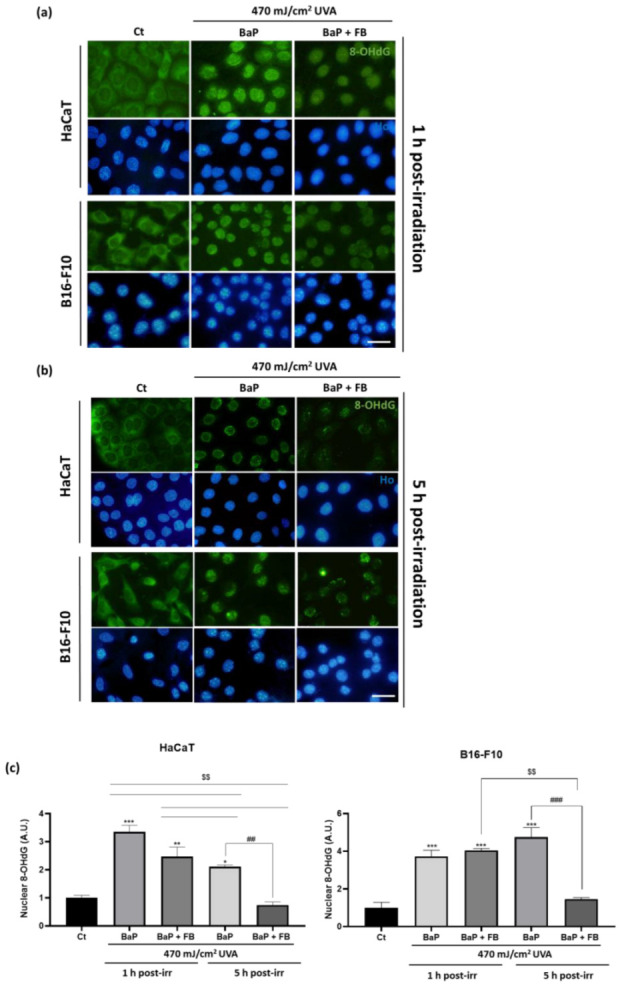
Localization and intensity of 8-OHdG in HaCaT and B16-F10 cells after BaP, FB and UVA light treatments. Cells incubated with BaP or BaP + FB and immediately exposed to 470 mJ/cm^2^ of UVA light. 8-OHdG expression was evaluated 1 h and 5 h after light exposure. (**a**) Fluorescence microscopy images of 8-OhdG localization 1 h after irradiation. Cells treated with BaP and exposed to UVA light showed increased 8-OHdG fluorescence levels at the nuclei of the cells. Cells treated with BaP + FB and sequentially exposed to UVA light showed a dramatic reduction of nuclei accumulation. (**b**) Fluorescence microscopy images of 8-OhdG 5 h after irradiation. All treatment conditions showed a decrease in fluorescence intensity inside cell nuclei compared with that observed 1 h after the treatments. Scale bar: 20 µm. (**c**) Quantification of 8-OhdG fluorescence intensity in both cell lines with the ImageJ Software. The results showed a significant increase in cells treated with BaP and exposed to UVA light in both 1 h and 5 h after irradiation. Moreover, intensity levels in cells treated with BaP + FB and UVA light were also significantly higher when compared to the control, but significantly slower when compared to BaP combined with UVA light 1 h after irradiation. 5 h after irradiation, intensity in all conditions decreased significantly in HaCaT cells, but not B16-F10 cells. In both cell lines, 8-OHdG levels in cells treated with FB + BaP decreased 5 h after irradiation. Error bars denote ± S.E.M. (*n* = 3, one-way ANOVA compared to Ct cells *: *p* < 0.05; **: *p* < 0.01; ***: *p* < 0.001; *n* = 3, *t* test compared to BaP treated cells ##: *p* < 0.01; ###: *p* < 0.001; and *n* = 3, *t*-test compared to 5 h post-irradiated cells $$: *p* < 0.01).

The 8-OHdG marker is routinely used as an indicator of oxidative stress and nucleic acid damage triggered by UV irradiation. Because of the fast capacity of cells to repair this oxidized derivative, short time points after UVA light exposure (1 h and 5 h) were selected to evaluate the presence of 8-OHdG by indirect immunofluorescence [44]. Untreated (control) cells displayed low basal levels of 8-OHdG mostly localized in the cytoplasm (Figure 7a), suggesting that nuclear DNA remains protected from oxidation in resting conditions. In response to single treatment with BaP or with UVA light, despite the nuclear levels of 8-OHdG were significantly increased 1 h after the treatment with either agent in both cell lines, they were completely reverted after 5 h in HaCaT cells, but not in B16-F10 cells (Figure S8). The combined treatment with BaP followed by UVA light irradiation induced abundant nuclear accumulation of 8-OHdG in both cell lines, suggesting profound genomic damage. The combined treatment with BaP followed by UVA light irradiation induced abundant nuclear accumulation of 8-OHdG in both cell lines, suggesting profound genomic damage. This result was particularly remarkable shortly after the treatment (1 h) (Figure 7a,c), indicating that nucleic acid oxidation occurs at early timepoints after the administration of the combined treatment. Interestingly, we observed a decrease in the nuclear levels of 8-OHdG with time (results are shown 5 h after treatment) (Figure 7b,c), especially in HaCaT cells, suggesting that rapidly activated DNA repair mechanisms could naturally contribute to partially repair the damage caused. Although partially reduced, 5 h after the sequential treatment nuclear levels of 8-OHdG still remained significantly higher than in the control in both cell lines. It is worth noting that a unique significant dramatic reduction of the nuclear 8-OHdG levels was only achieved in cells treated with FB together with BaP prior to UVA light irradiation, reaching values non significantly different than those of the control (Figure 7b,c). This suggests that FB can prime the cells and foster the DNA repair mechanisms operating to efficiently replace oxidized nucleic acids that appear as a consequence of the sequential treatment with BaP and UVA light.

### 3.5. Effect on the Expression of Opsin-3 in B16-F10 Cells

**Figure 8:**
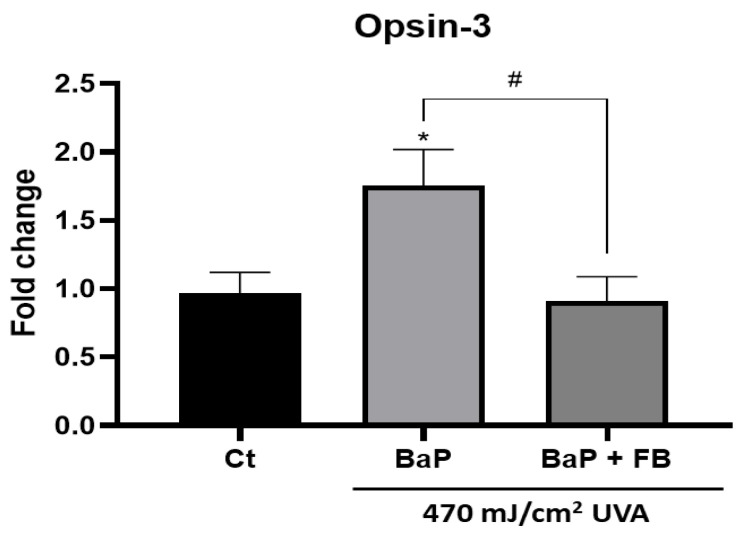
Opsin-3 expression in non-irradiated B16-F10 cells and cells loaded with BaP, FB and exposed to UVA light. Cells were treated with BaP and BaP + FB and subsequently irradiated with 470 mJ/cm^2^ of UVA light. Opsin-3 was quantified 24 h after irradiation through RT-PCR. Opsin-3 expression appeared to increase in cells treated with BaP, whereas FB treatment diminished this expression reaching control values. (*n* = 3, one-way ANOVA compared to Ct cells *: *p* < 0.05 and *n* = 3, *t*-test compared to BaP treated cells #: *p* < 0.05).

Melanosomes are the cytoplasmic organelles in which melanogenesis takes place. As their presence is characteristic of melanocytes, we specifically used the B16-F10 melanocyte cell line to assess the expression of Opsin 3, a protein that is directly related to melanogenic cellular pathways. Opsin-3 expression levels were quantified by RT-PCR in non-irradiated cells and in cells pre-treated with BaP alone or in combination with FB and exposed to UVA light. Opsin-3 has been shown to be related to melanogenesis increase processes. The results obtained showed a significant increase in the Opsin-3 expression in cells treated with BaP and then exposed to UVA light, whereas cells treated with the combination of BaP and FB prior to UVA irradiation diminished such expression reaching control values (Figure 8). Single treatments did not produce changes in Opsin-3 expression (Figure S9).

## 4. Discussion

It is a fact that the BaP air pollutant, also present in tobacco smoke and in different foods, produces negative effects in the population. Among these effects, ROS generation and DNA damage stand out. Moreover, previous works have pointed to a deleterious synergy of BaP and solar radiation, specifically its UVA component [15,16]. Although this phenomenon has been addressed in numerous studies, not much is known about the combined effect of these two agents in cells derived from melanoma or from adult skin [45]. Thus, the present work primarily analyzes the response of B16-F10 and HaCaT cells to the sequential treatment with BaP and UVA light.

In addition, based on previous studies, FB is a promising photoprotector, specially preventing oxidative stress and DNA damage [39,46]. *Polypodium leucotomos*’ extract has been extensively used, mostly to counteract oxidative stress. While oral and systemic administration strategies have been used to modulate immune and inflammatory responses triggered by UVB irradiation, topical administration has been chosen to study photoprotection as well as for the treatment of skin-related pathologies such as atopic dermatitis and psoriasis [36,47,48]. Consequently, topical administration of *Polypodium leucotomos*’ extract is the via of administration more representative in the context of our study. In this line of work, the photoprotective role of FB against the combination of BaP and UVA light has also been evaluated.

Our results indicated that single treatment with low doses of UVA light (up to 1 J/cm^2^) was not harmful, in agreement with previously published results [49]. Equally, BaP alone remained non-toxic at the concentrations selected for this study (2 µM for HaCaT cells, and 5 µM for B16-F10 cells), matching previous results generated in liver cells [50,51]. Importantly, we demonstrated that the sequential treatment with these non-toxic doses of BaP particles and UVA light did induce a significant decrease in cell viability in both cell lines, confirming the deleterious synergistic effect anticipated by other authors [52,53]. However, this outcome was significantly improved when FB was added to the cells during the incubation with BaP prior to UVA light exposure, supporting a photoprotective effect of FB [34].

As other authors have reported increased ROS production in response to the combination of BaP and UVA light in epidermoid carcinoma cells [53], we evaluated the level of oxidative stress in both cell lines. Indeed, we found significantly higher ROS levels upon sequential treatment with BaP and UVA light, while single treatment with either BaP or UVA light did not alter ROS production in comparison to control cells. The deleterious synergistic effect was again effectively counteracted by the addition of FB during the incubation with BaP prior to UVA light exposure. In both cell lines, FB induced a significant reduction of ROS levels compared to the treatment with BaP + UVA light in the absence of FB, although in B16-F10 cells the ROS levels remained significantly increased compared to the control. These results are in agreement with previous data [46] and support a protective effect of FB to prevent excessive oxidative stress.

The exposure to high doses of BaP has been associated by Jiang et al. with the induction of mitochondrial dysfunction, using a genetically altered model of Hep3B cells [51]. Our results revealed morphological alterations in mitochondria and cytochrome C release as a consequence of the sequential treatment with BaP and UVA light. The addition of FB during the incubation with BaP prior to irradiation with UVA light not only prevented the morphological changes in mitochondria, but also partially rescued the liberation of cytochrome C into the cytoplasm. Similar results were previously reported by our group when evaluating the potential of FB to prevent blue light-induced cell damage [46].

Two classical markers, γH2AX and 8-OHdG, were used in this study to address the induction of DNA damage. According to previous results by Toyooka et al. [20], the expression of yH2AX was detected in a significantly higher proportion of cells after exposure to BaP followed by irradiation with UVA light in both cell lines. The addition of FB during the incubation with BaP prior to UVA light exposure significantly reduced the percentage of γH2AX positive nuclei in both cell lines, albeit HaCaT cells still retained significantly higher levels than the control. Regarding 8-OHdG, as repair mechanisms are activated shortly after the induction of DNA damage [44], we decided to evaluate the presence of this marker at short time points (1 h and 5 h post-treatment). Our results revealed increased nuclear levels of this oxidized derivative 1 h after the treatment with BaP followed by UVA light, suggesting relevant oxidative genomic damage, which is in agreement with previous works in mammalian cells [54]. Supporting the idea that this striking effect was derived from the deleterious synergistic action of the combination of both agents, we observed that single treatment with either BaP or UVA light in HaCaT cells did induce a significant increase in the levels of 8-OHdG at short timepoints (1 h), but this effect was not maintained, as no differences were detected 5 h after the treatments. This is in agreement with the expected rapid elimination of this oxidized derivative from the nuclear DNA [44]. Importantly, the increase of 8-OHdG nuclear levels driven by the treatment with BaP + UVA light was significant both 1 h and 5 h after treatment, compared to non-treated control cells. In contrast, the addition of FB together with BaP prior to the irradiation with UVA light led to a unique dramatic reduction of the signal, achieving final levels of nuclear 8-OHdG that did not significantly differ from those of the control. Altogether, these results strongly support that FB induces the priming of the cells that become prompted to rapidly activate repair mechanisms, leading to the efficient elimination of oxidized derivatives that appear in the nuclear DNA as a consequence of the sequential exposure to BaP and UVA light.

Finally, as other authors have already addressed, UVA light influences melanogenesis [25,26,27]. To deepen into the mechanisms underlying the effect of BaP on melanogenesis, the mouse-derived B16-F10 cell line has been selected as it is an exceptional and extensively used in vitro model in the field of cancer and melanogenesis, considered representative of physiological and pathological mechanisms operating in human cells [55]. The treatment with BaP + UVA light induced a significant increase in the expression of Opsin-3 in B16-F10. Nonetheless, this effect was reversed when cells were additionally treated with FB during the incubation with BaP prior to UVA light exposure. The protective effect of FB against melanogenesis reported in this work is in agreement with previous studies published by our group. Thus, we provide direct evidence of the photoprotective role exerted by FB, not only by preventing cell damage induced by light irradiation—as previously published by our group [46]—but also by preventing melanogenesis activation induced by the sequential treatment with BaP and UVA light through the modulation of opsin-3 expression.

## 5. Conclusions

In sum, our results support the idea that sequential treatment with BaP and UVA light can exert a deleterious synergistic effect in the skin by inducing increased oxidative stress and DNA damage, as well as by promoting the overexpression of the opsin-3 photoreceptor. Importantly, we have proven that FB can prevent these effects, emerging as a highly potent agent against environmental insults such as the air pollution and sun irradiation.

## Data Availability

Data is contained within the article or supplementary material.

## Supplementary Materials

The following supporting information can be downloaded at: https://www.mdpi.com/article/10.3390/antiox11112185/s1, Figure S1. Effect of different doses of UVA radiation and BaP alone in HaCaT and B16-F10 cells; Figure S2. Effect of different doses of Fernblock in combination with the selected BaP doses for each cell line; Figure S3. Effects of BaP, FB and UVA light exposure on the viability and morphology of HaCaT and B16-F10 cell lines; Figure S4. Reactive oxygen species generation in HaCaT and B16-F10 cells with single treatments of BaP and UVA light; Figure S5. Mitochondrial morphology in HaCaT and B16-F10 non-treated cells and BaP and UVA light single treatments; Figure S6. Effect of BaP and UVA light single treatments on the dynamics of cytochrome C in HaCaT and B16-F10 cells; Figure S7. γH2AX localization and positiveness in HaCaT and B16-F10 cells after single BaP and UVA light treatments; Figure S8. Localization and intensity of 8-OHdG in HaCaT and B16-F10 cells after BaP and UVA light single treatments; Figure S9. Opsin-3 expression in non-irradiated B16-F10 cells and cells with BaP or UVA light single treatments.
