# Supplementary material for: Protective Effect of the Hydrophilic Extract of Polypodium leucotomos, Fernblock®, against the Synergistic Action of UVA Radiation and Benzo[a]pyrene Pollutant
Source: Antioxidants (Basel). 2022 Nov 4;11(11):2185. doi: 10.3390/antiox11112185 (PMC9686834; doi:10.3390/antiox11112185)
Supplement: Supplementary file 1 [file antioxidants-11-02185-s001.zip › antioxidants-1988056-supplementary.pdf]

antioxidants

MDPI

Protective effects of the hydrophilic extract of Polypodium leucotomos, Fernblock®, against the synergistic action of UVA radiation and benzo[a]pyrene pollutant

Supplementary materials

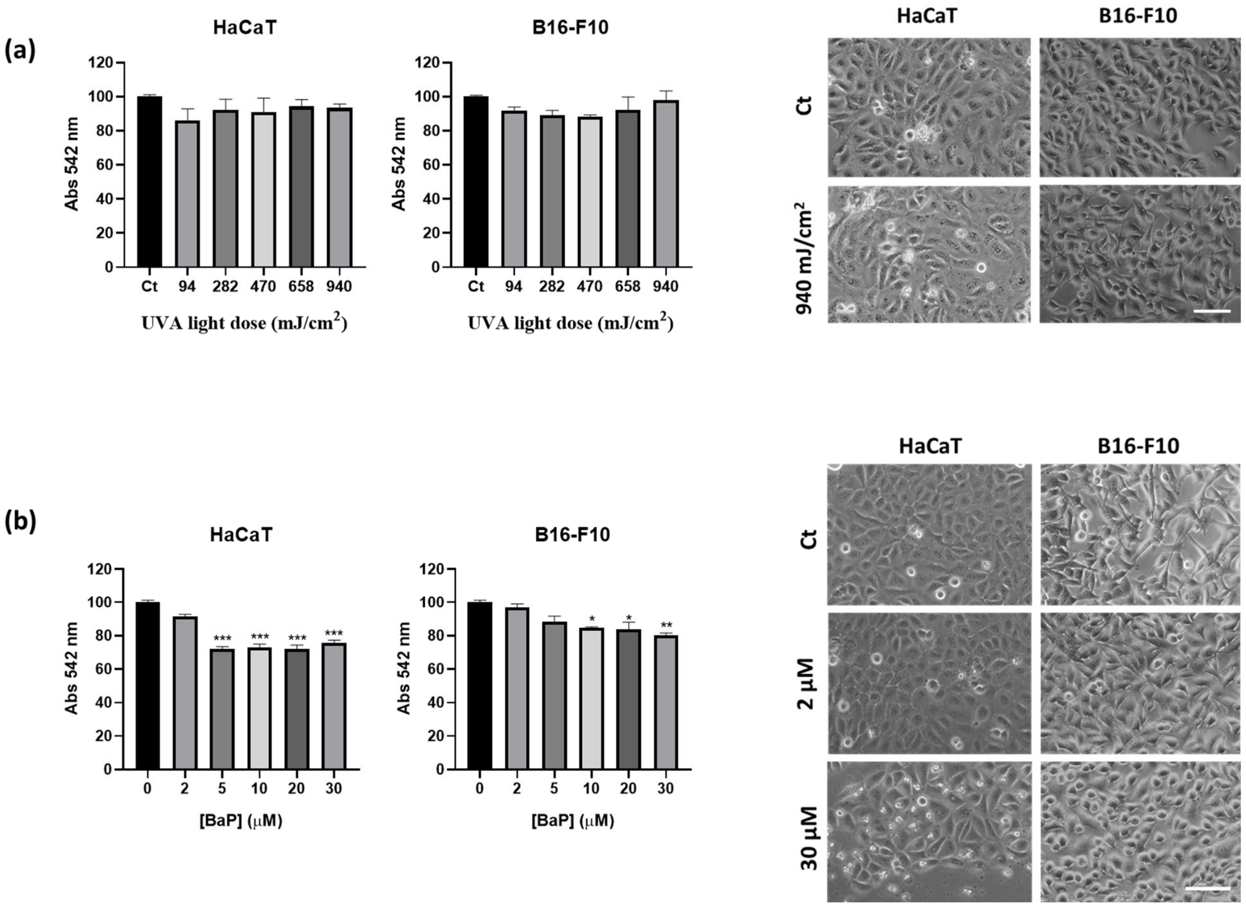

Figure S1. Effect of different doses of UVA radiation and BaP alone in HaCaT and B16-F10 cells. (a) Cell survival represented in percentage of the effect of UVA light in both cell lines and representative phase contrast images. In any of the cases, UVA light alone induced damage to the cells. Scale bar = 50 μm. (b) Cell survival represented in percentage of the effect of BaP alone in both cell lines and representative phase contrast images. HaCaT cells were more sensitive to BaP, with a significant decrease in cell survival from 5 μM onwards. B16-F10 cells survival significantly decreased with 10 μM onwards. Error bars denote ± S.E.M. (n=3, one-way ANOVA *: $p<0.05$; **: $p<0.01$; ***: $p<0.001$. Scale bar = 50 μm.

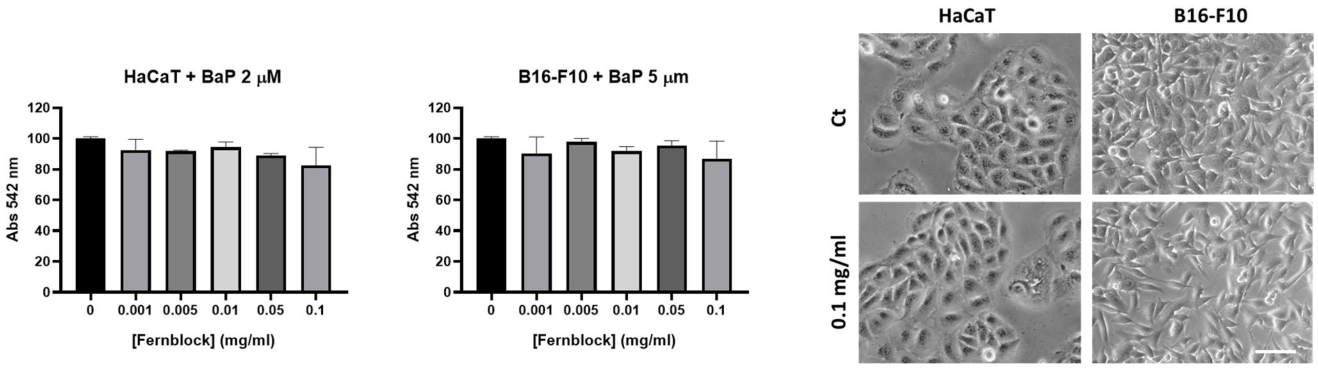

Figure S2. Effect of different doses of Fernblock in combination with the selected BaP doses for each cell line. Cell survival is represented in percentage. In both cell lines, the combination of BaP and Fernblock without UVA light did not induce cell damage. Error bars denote ± S.E.M. Representative phase contrast images illustrate these results with the higher Fernblock dose. Scale bar = 50 μm.

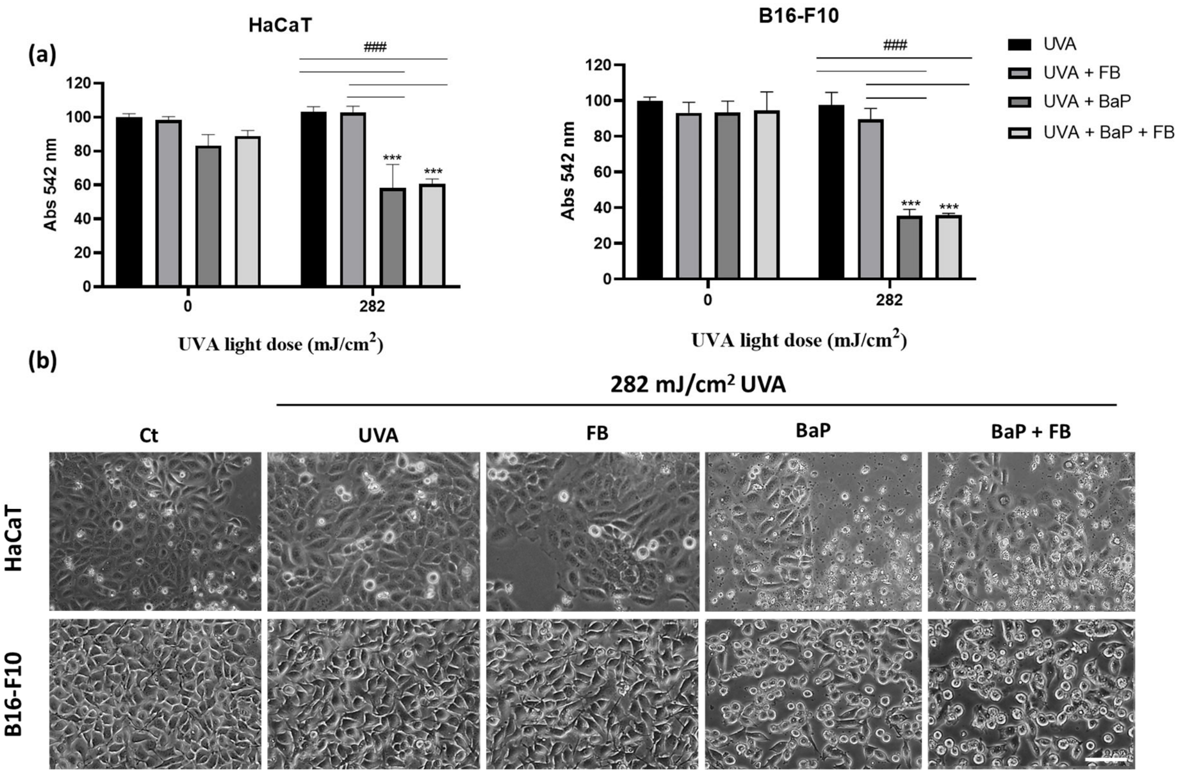

Figure S3. Effects of BaP, FB and UVA light exposure on the viability and morphology of HaCaT and B16-F10 cell lines. (a) Cell viability rates of both cell lines treated with BaP (2 μM, HaCaT; 5 μM, B16-F10), FB (0.01 mg/mL) and exposed to 282 mJ/cm² of UVA light. The results did not show significant differences in cell viability between BaP + UVA light and BaP + FB + UVA light in both cell lines. (b) Phase-contrast microscopy images illustrating the changes in cell morphology after the treatments. Error bars denote ± S.E.M. (n=3, one-way ANOVA ***: $p<0.001$). Scale bar = 50 μm.

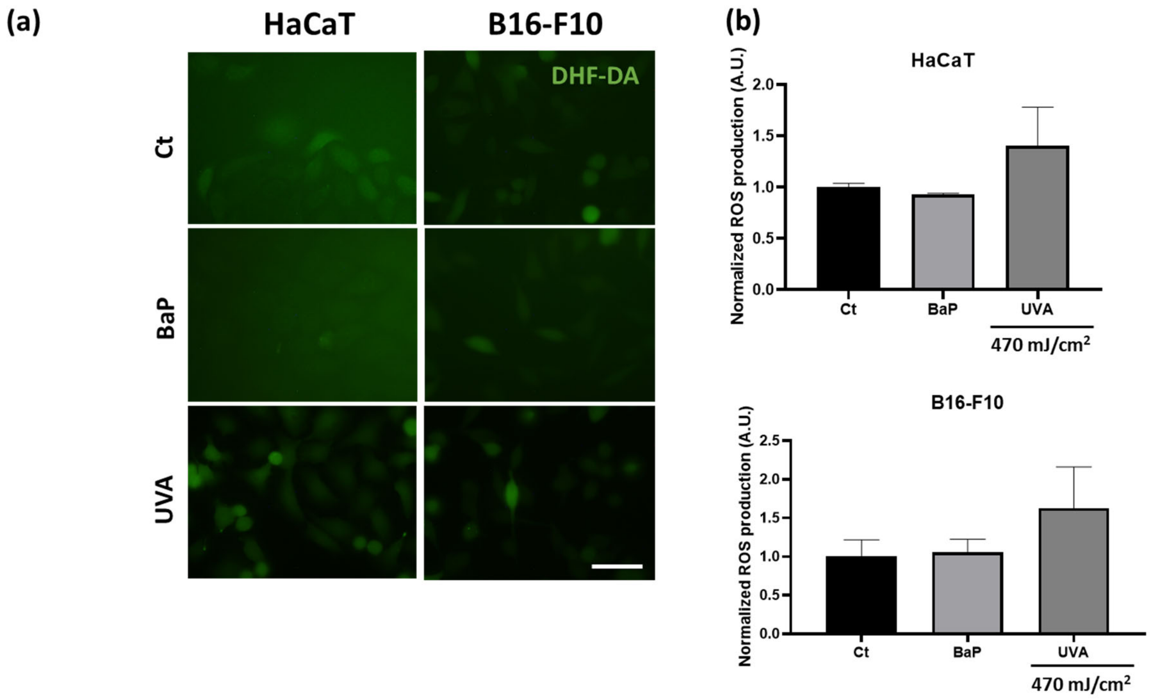

Figure S4. Reactive oxygen species generation in HaCaT and B16-F10 cells with single treatments of BaP and UVA light. (a) ROS production images in non-treated and BaP or UVA single treatment cells. No differences in ROS generation could be observed. Scale bar: 40 µm. (b) ROS production was quantified using the Image J Software. There were no significant differences between control cells and single treatments in both cell lines. Error bars denote ± S.E.M.

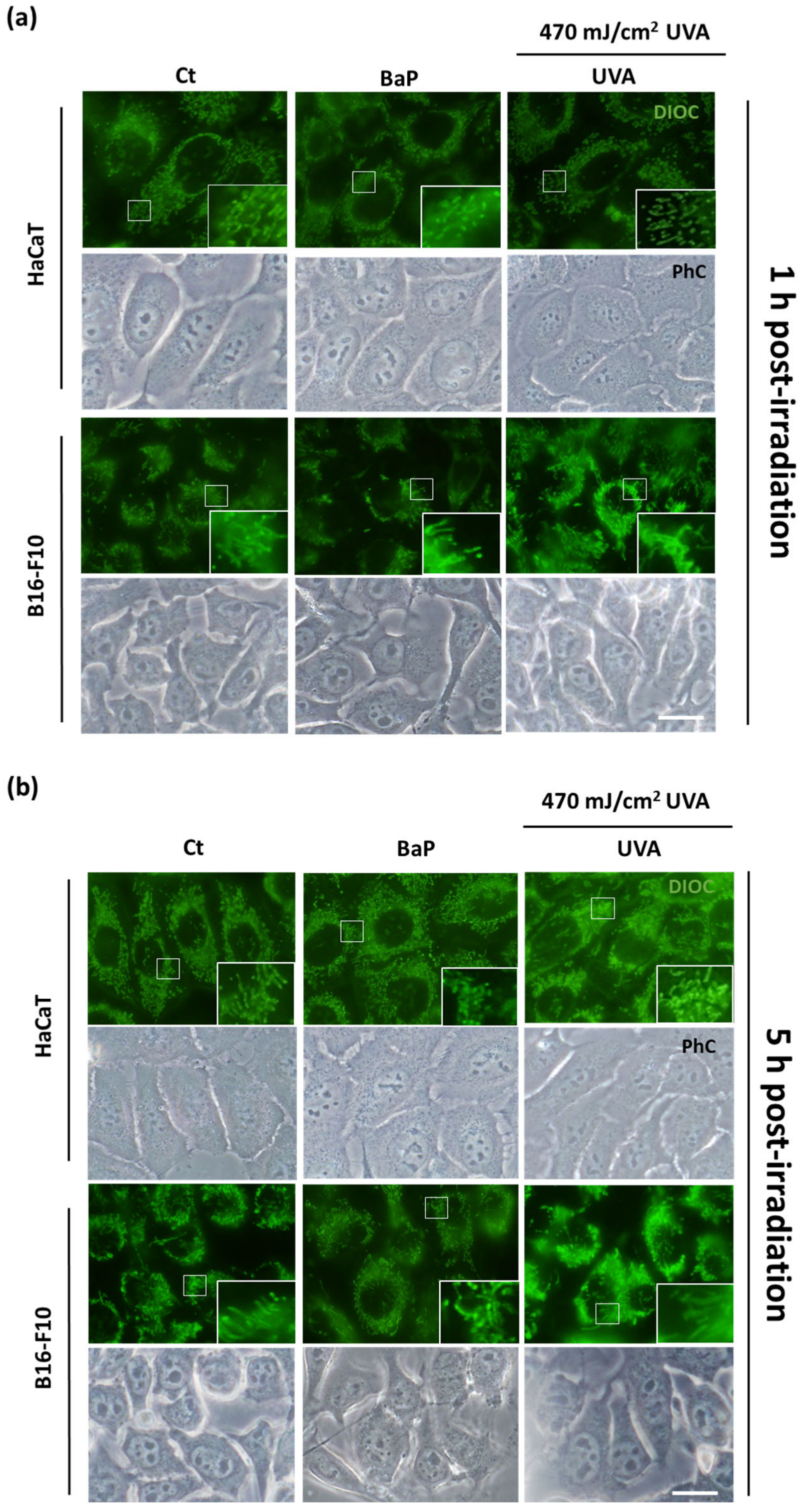

Figure S5. Mitochondrial morphology in HaCaT and B16-F10 non-treated cells and BaP and UVA light single treatments. (a) Mitochondrial morphology 1 h after irradiation. No differences in cell morphology between controls could be observed in both cell lines. (b) Mitochondrial morphology 5 h after irradiation. In both cell lines, cells did not exert morphological changes with any of the treatments. Phase contrast images did not show morphological changes in cell shape. Scale bar: 20 μm.

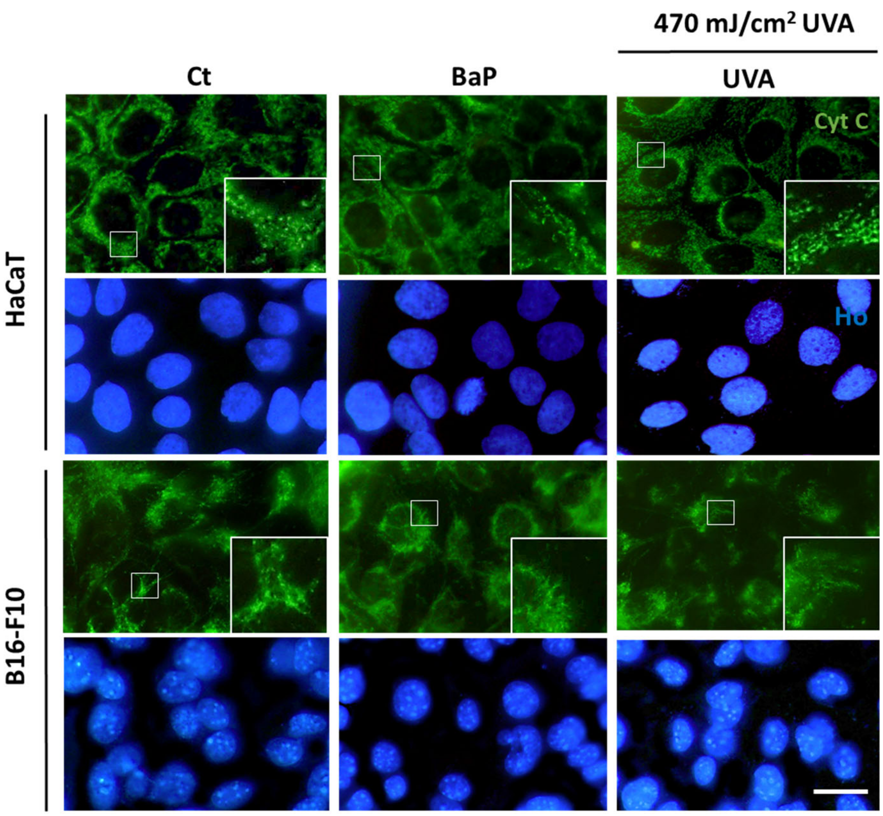

Figure S6. Effect of BaP and UVA light single treatments on the dynamics of cytochrome C in HaCaT and B16-F10 cells. The results showed no altered mitochondrial morphology or cytochrome C release in cells treated with single treatments in both cell lines. Scale bar: 20 µm.

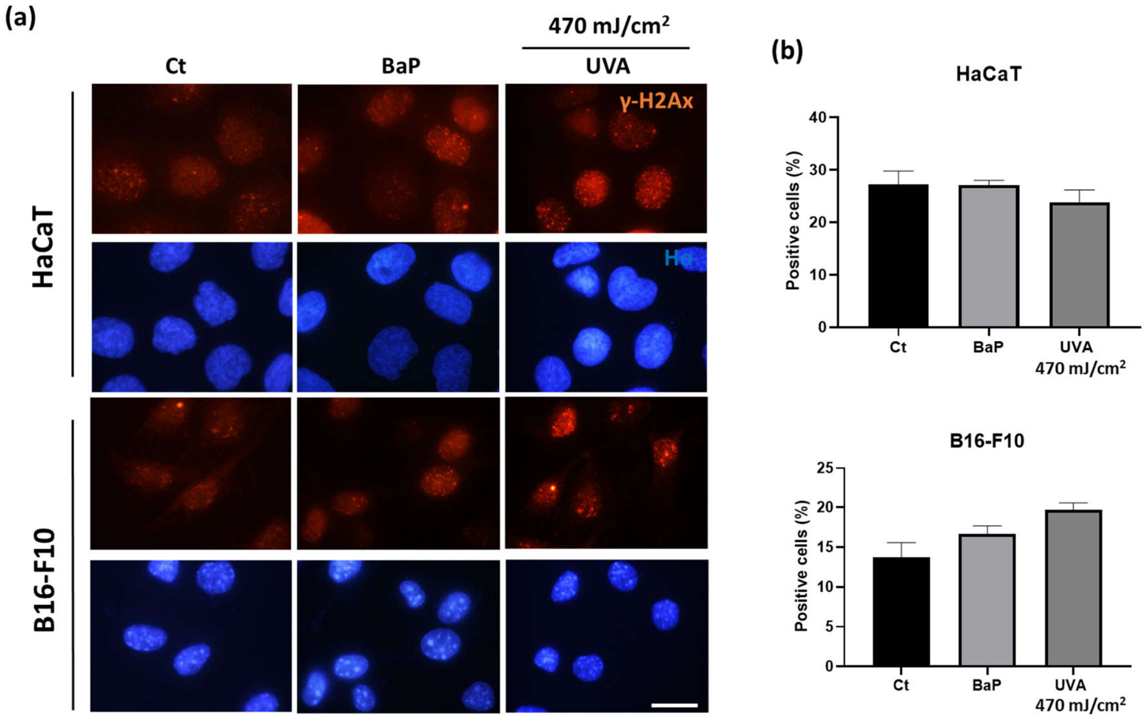

Figure S7. γH2AX localization and positiveness in HaCaT and B16-F10 cells after single BaP and UVA light treatments. (a) Fluorescence microscopy images of positive and negative γH2AX cells. Cells treated with BaP alone or UVA light alone displayed basal γH2AX expression levels. Scale bar: 20 µm. (b) Quantification of positive γH2AX cells in both cell lines. The results showed no significant

differences between single treatments and untreated cells in both cell lines. Error bars denote ± S.E.M.

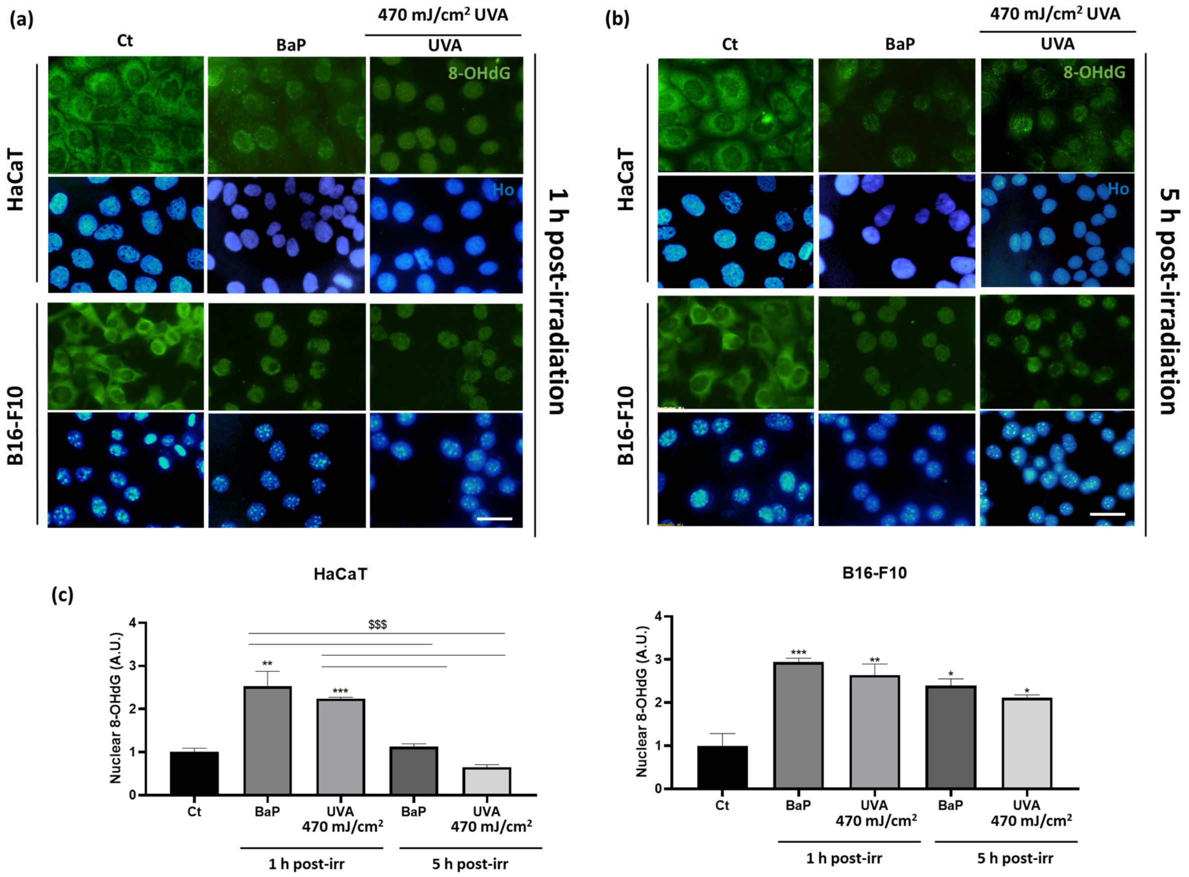

Figure S8. Localization and intensity of 8-OHdG in HaCaT and B16-F10 cells after BaP and UVA light single treatments. (a) Fluorescence microscopy images of 8-OHdG localization 1 h after irradiation. Cells treated with BaP or UVA light showed increased 8-OHdG fluorescence levels at the nuclei of the cells. (b) Fluorescence microscopy images of 8-OhdG 5 h after irradiation. All treatment conditions showed a decrease in fluorescence intensity inside cell nuclei compared with that observed 1 h after the treatments in HaCaT cells, but no B16-F10. Scale bar: 20 µm. (c) Quantification of 8-OhdG fluorescence intensity in both cell lines with the ImageJ Software. The results showed a significant increase of nuclei signal intensity in cells treated with BaP or UVA light only in the case of 1 h after irradiation in HaCaT cells, but it showed a significant increase 1h and 5 h after irradiation in B16-F10 cells. Error bars denote ± S.E.M. (n=3, one-way ANOVA compared to Ct cells *: $p<0.05$; **: $p<0.01$; ***: $p<0.001$; n=3, t test compared to BaP treated cells #: $p<0.05$; ##: $p<0.01$; ###: $p<0.001$; and n=3, t test compared to 5 h post-irradiated cells $$$: $p<0.001$).

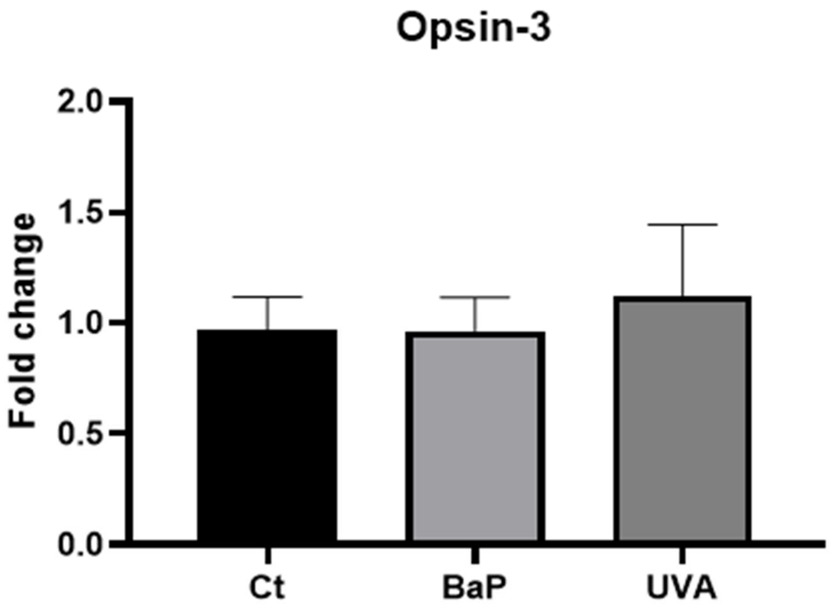

Figure S9. Opsin-3 expression in non-irradiated B16-F10 cells and cells with BaP or UVA light single treatments. Opsin-3 expression did not show significant differences when exposed to single treatments. Error bars denote ± S.E.M.
